# Prolonged alpha-blockade and doxazosin are associated with hypertensive crisis in pheochromocytoma surgery

**DOI:** 10.3389/fendo.2025.1682912

**Published:** 2026-01-07

**Authors:** Karolina Zawadzka, Magdalena Pisarska-Adamczyk, Alicja Hubalewska-Dydejczyk, Michał Pędziwiatr

**Affiliations:** 1 Doctoral School of Medical and Health Sciences, Jagiellonian University Medical College, Krakow, Poland; 2 Department of General Surgery, Jagiellonian University Medical College, Krakow, Poland; 3 Department of Medical Education, Jagiellonian University Medical College, Krakow, Poland; 4 Chair and Department of Endocrinology, Jagiellonian University Medical College, Krakow, Poland

**Keywords:** pheochromocytoma, hypertensive crisis, pretreatment, alpha-blockade, selective, non-selective

## Abstract

**Objective:**

Hypertensive crisis represents a significant intraoperative challenge in pheochromocytoma surgery, often necessitating immediate pharmacologic intervention. To reduce this risk, preoperative α-adrenergic blockade is routinely implemented. Although prior studies have addressed the choice of α-blockade, the impact of its type, treatment duration, and final titrated dose on hypertensive crisis has not yet been established.

**Methods:**

A retrospective analysis was conducted on 110 patients undergoing laparoscopic adrenalectomy for pheochromocytoma. The type of alpha-blocker (doxazosin: DOX vs. phenoxybenzamine: PXB), duration of preoperative preparation, and final titrated dose were evaluated regarding their impact on the risk and duration of intraoperative hypertensive crisis (SBP >200 mmHg).

**Results:**

The duration of hypertensive crisis was significantly longer in the DOX group (median 15.0 (10.0-30.0) vs. 10.0 (5.0-15.0 min, p=0.03). The DOX group demonstrated higher intraoperative vasopressor use (39.6% vs. 10.9%, p<0.001), and a longer perioperative hospitalization compared to the PXB group (median 3.0 (2.2-4.0) vs 2.0 (2.0-3.0), p<0.001). Diabetes, urinary metanephrines >10× ULN, and preoperative α-blockade >30 days were independent risk factors for hypertensive crisis. Prolonged blockade was linked to longer crisis duration and increased vasopressor use, particularly with DOX. The final α-blocker dose did not influence hypertensive outcomes.

**Conclusion:**

Selective α-blockade with doxazosin resulted in longer hypertensive crises, increased intraoperative vasopressor requirements, and prolonged hospitalization. Prolonged α-blockade (>30 days) was associated with more frequent and prolonged hypertensive crises and a higher risk of postoperative vasopressor use.

## 1 Introduction

Pheochromocytomas (PHEO) are neuroendocrine tumours originating from chromaffin cells of the adrenal medulla. Surgical resection is the treatment of choice; however, it carries a high risk of severe perioperative hemodynamic instability due to catecholamine surges during anaesthesia or tumour manipulation. To minimise these complications, preoperative alpha-adrenergic blockade remains the gold standard in preparation for adrenalectomy for PHEO (1, 2).

During surgery, specific systolic blood pressure (SBP) thresholds are recognized as critical indicators of potential complications. SBP above 160 mmHg are generally considered moderate hypertension and warrant close monitoring, particularly in patients with pre-existing cardiovascular disease (3). A systolic pressure of 180 mmHg is typically regarded as a critical threshold, where the likelihood of serious complications, such as myocardial infarction, stroke, aortic dissection, and heart failure, becomes considerably higher (4). However, the most severe risk is observed when SBP surpasses 200 mmHg, as this is associated with the highest likelihood of life-threatening complications, including aneurysm rupture, haemorrhagic stroke, and acute coronary events (5, 6). In the context of pheochromocytoma surgery, this threshold is widely recognised in the literature as a critical point beyond which the risk of severe hemodynamic complications increases significantly (7–10).

Although previous research has already compared the efficacy of these two types of blockade, most of them focused on SBP thresholds above 160 mm Hg (11, 12), and the optimal duration and final dose of blockade remain undefined. Recent evidence suggests that extending the duration of preoperative alpha-blockade may not provide additional benefit in preventing intraoperative hemodynamic instability (13, 14). Furthermore, prolonged pretreatment may increase the risk of postoperative hypotension requiring vasopressor support (14). Recent findings also suggest that a non-escalating dose strategy may offer comparable intraoperative blood pressure control while reducing the risk of postoperative hypotension and shortening hospital stay (15). In light of these findings, we aimed to assess whether the type of alpha-blockade administered (doxazosin vs. phenoxybenzamine), the duration of preoperative preparation, and the final titrated dose influence the risk and duration of intraoperative hypertensive crisis (defined as SBP >200 mm Hg), as well as the occurrence of postoperative hypotension requiring vasopressor therapy.

## 2 Materials and methods

### 2.1 Inclusion and exclusion criteria

We conducted a retrospective observational study to evaluate how the type of alpha-blockade, the duration of preoperative treatment, and the final titrated dose affect intraoperative hemodynamic parameters, particularly the occurrence and duration of hypertensive crises as well as perioperative outcomes including the use of vasoactive drugs, operative time, and postoperative complications, in patients undergoing laparoscopic adrenalectomy for pheochromocytoma. Patients diagnosed with PHEO who underwent laparoscopic adrenalectomy from 2003 to 2022 were included. Inclusion criteria comprised postoperative histopathological diagnosis of PHEO and complete perioperative hemodynamic data. Exclusion criteria involved patients with metastatic disease at the diagnosis, bilateral pheochromocytomas, incomplete data, or cases converted to open surgery. Patients whose post-operative pathology examination revealed the presence of PHEO tissue, but were referred for surgery as incidentalomas (without detectable hormonal activity) and were not pretreated with doxazosin or phenoxybenzamine, were also not included in this study.

### 2.2 Preparation for surgery

A preoperative diagnosis of PHEO was made in the endocrinology department. Preoperatively, all adrenal tumours were assessed by imaging studies, namely computed tomography (CT) and/or magnetic resonance imaging. A routine panel of laboratory tests was performed prior to surgery to determine the hormonal activity of the tumour. The hormonal evaluation included metoxycatecholamines’ metabolites measurement (metanephrine, normetanephrine, and methoxytyramine) from 24-hour urine collection along with an overnight 1-mg dexamethasone suppression test.

All patients began preoperative treatment with either phenoxybenzamine, an irreversible, non-competitive alpha-blocker, or doxazosin, a selective alpha-blocker. Doses were titrated based on clinical response. Patients operated between 2003 and 2011 received phenoxybenzamine as the standard preparation, while from 2012 onwards doxazosin was used due to changes in drug availability and institutional practice, reflecting the broader shift in prescribing patterns across Europe. Moreover, when tachycardia coexisted, the administration of beta-blockers was implemented.

### 2.3 Surgery

Adrenalectomies were performed in a tertiary centre with extensive expertise in adrenal surgery. The preferred surgical technique was laparoscopic transperitoneal lateral adrenalectomy, described in detail elsewhere (16, 17). The main adrenal vein was isolated and clipped prior to mobilizing the adrenal gland. Anaesthetic management was based on established peri-operative principles for pheochromocytoma surgery described in the published literature and was applied consistently in our centre (1). Invasive arterial monitoring was established before induction, sympathomimetic agents were avoided, and short-acting vasoactive agents were prepared in advance. Vasoactive medications were administered in response to specific haemodynamic indications. Short-acting vasodilators and beta-blockers were used during tumour manipulation to treat catecholamine-induced hypertension and tachycardia, particularly during pneumoperitoneum, dissection of the adrenal gland and manipulation of adrenal venous outflow. Abrupt rises in systolic blood pressure were managed with titrated boluses or continuous infusions of vasodilators such as sodium nitroprusside or urapidil, while beta-blockers were used when tachyarrhythmias developed. Following adrenal vein ligation, vasopressors such as norepinephrine were commenced to support blood pressure in the setting of catecholamine withdrawal, vasodilatation and circulatory instability. Postoperative vasopressor use was recorded for all patients. Any administration of vasopressors in the post-anaesthesia care unit (PACU) and/or in the intensive care unit (ICU) was registered, regardless of duration.

### 2.4 Outcomes

The primary endpoint of the study was the occurrence of intraoperative hypertensive crisis, defined as any episode of systolic blood pressure (SBP) exceeding 200 mmHg and sustained for more than one minute. This threshold and time criterion were adopted based on evidence demonstrating that SBP above 200 mmHg are associated with a markedly increased risk of life-threatening cardiovascular complications, such as myocardial infarction, haemorrhagic stroke, and aortic dissection (5, 6). In the setting of pheochromocytoma surgery, this definition reflects clinically meaningful hemodynamic instability and is consistent with thresholds used in prior literature (7–10). Intraoperative blood pressure was monitored at 5-minute intervals and documented continuously. Secondary outcomes included the cumulative duration of hypertensive crises, intraoperative administration of vasodilators and vasopressors, postoperative requirement for vasopressor support, hospital length of stay, and the occurrence of postoperative complications. Complications were graded according to the Clavien–Dindo classification system, with severe complications defined as grade 3 or higher. Mortality occurring during hospitalisation or within 30 days postoperatively was also recorded.

### 2.5 Data collection

Data were collected retrospectively from both electronic medical records and manually recorded perioperative documentation. Baseline characteristics included age, sex, body mass index (BMI), and comorbid conditions such as diabetes mellitus, hypertension, coronary artery disease, chronic kidney disease, and history of previous abdominal surgery. The physical status of each patient was evaluated preoperatively using the American Society of Anaesthesiologists (ASA) classification. Tumour characteristics, including laterality and size, were determined based on preoperative imaging (CT or MRI), and hormonal activity was assessed based on 24-hour urinary excretion of metoxycatecholamine metabolites (metanephrine, normetanephrine, and 3-methoxytyramine) measured via high-performance liquid chromatography. Pharmacological preparation details encompassed the type and dose of alpha-blocker (doxazosin or phenoxybenzamine), duration of pretreatment, and concurrent use of beta-blockers or other antihypertensive medications. Intraoperative data included duration of surgery and anaesthesia, estimated blood loss, volume of fluids administered, and use of vasoactive agents. Postoperative data included vasopressor requirement, complications, and length of hospital stay. All records were reviewed independently by two investigators (KZ and MPA) for accuracy and reliability.

### 2.6 Statistical analysis

Descriptive statistics were applied to characterize the study cohort. Descriptive statistics included frequency and percentage for nominal variables and mean ± standard deviation (SD) or as median and interquartile range (IQR) for continuous variables. Continuous variables were analysed using t-tests or Mann-Whitney U tests, depending on the distribution normality, while categorical variables were assessed with chi-square (χ²) tests. Univariable logistic regression was used to identify factors associated with intraoperative hypertensive crises, defined as systolic blood pressure >200 mmHg. Variables with a p-value <0.10 in univariable analysis were included in the multivariable logistic regression model. Associations between predictors and outcomes were quantified using odds ratios (OR) along with their corresponding 95% confidence intervals (CI). Additionally, Spearman’s rank correlation coefficients were calculated to explore the relationships between duration and dose of alpha-blockade and perioperative hemodynamic outcomes. Statistical significance was set at a P-value below 0.05. All statistical analyses were conducted using Statistica software (version 13, StatSoft Inc.) and the R programming environment (version 4.3.2; R Foundation for Statistical Computing).

## 3 Results

The analysis included 110 patients with histopathologically confirmed pheochromocytoma. The median age was 55 years (IQR: 42–65), and the median tumour size was 3.2 cm (IQR: 2.5–4.5) (**Table 1**). All procedures were intended to be performed laparoscopically; conversion to open surgery was required in 3 patients (2.73%). The median duration of preoperative preparation was 44 days (IQR: 25.0–68.0) for patients receiving doxazosin and 21 days (IQR: 15.0–30.0) for those treated with phenoxybenzamine, p = 0.0065 (**Table 1**). The median titrated dose was 11 mg (IQR: 8.0–13.0) for doxazosin and 30 mg (IQR: 25.0–54.0) for phenoxybenzamine. The proportion of patients who received preoperative antihypertensive medications other than alpha-blockers did not differ significantly (**Table 1**).

**Table 1:** Patient demographics and tumour characteristics.

Variable	DOX (n=55)	PXB (n=55)	p
Age (years), median (IQR)	51 (46-70)	55 (37-65)	0.22
BMI (kg/m2), median (IQR)	25.9 (23.1-28.6)	24.6 (21.8-27.1)	0.21
Females, n (%)	27 (49.1%)	32 (58.2%)	0.44
Preoperative comorbidities, n (%)	37 (67.3%)	45 (81.8%)	0.13
Diabetes, n (%)	22 (40.0%)	19 (34.5%)	0.69
Hypertension, n (%)	30 (54.5%)	36 (65.5%)	0.33
Coronary artery disease, n (%)	10 (18.2%)	18 (32.7%)	0.13
Chronic obstructive pulmonary disease, n (%)	1 (1.8%)	5 (9.1%)	0.21
Chronic kidney disease, n (%)	3 (5.5%)	4 (7.3%)	1.00
Previous abdominal surgery, n (%)	12 (41.4%)	20 (37.0%)	0.88
ASA 1, n (%)	1 (1.8%)	1 (1.8%)	1.00
ASA 2, n (%)	31 (56.4%)	23 (41.8%)	0.19
ASA 3, n (%)	22 (40.0%)	29 (52.7%)	0.25
ASA 4, n (%)	1 (1.8%)	2 (3.6%)	1.00
Hereditary syndrome, n (%)	10 (18.9%)	5 (9.1%)	0.24
Right-side tumour, n (%)	31 (56.4%)	29 (52.7%)	0.85
Tumour size (cm), median (IQR)	3.75 (2.85–4.5)	3.0 (2.5–4.0)	0.23
PASS score, median (IQR)	3.0 (2.0-6.0)	4.0 (2.0-4.0)	0.40
Urine metanephrine (µl/24h), median (IQR)	1058 (432-2814)	2019 (742-4382)	0.11
Urine normetanephrine (µl/24h), median (IQR)	2098 (888-4471)	2581 (1508-6741)	0.20
Urine methoxytyramine (µl/24h), median (IQR)	396 (210-570)	388 (268-453)	0.87
Adrenergic phenotype, n (%)	3 (6.4%)	1 (3.8%)	1.00
Noradrenergic phenotype, n (%)	20 (42.6%)	9 (34.6%)	0.68
Mixed phenotype, n (%)	22 (46.8%)	16 (61.5%)	0.34
Use of β-blockers before surgery, n (%)	31 (62.0%)	17 (73.9%)	0.47
Use of other antihypertensive drugs before surgery, n (%)	19 (38.8%)	9 (39.1%)	1.00
Use of ACE inhibitors or ARBs before surgery, n (%)	11 (22.9%)	3 (13.0%)	0.53
Use of CCBs before surgery, n (%)	10 (20.8%)	4 (17.4%)	1.00
Use of diuretics before surgery, n (%)	9 (18.8%)	4 (17.4%)	1.00
Use of central-acting agents (clonidine) before surgery, n (%)	1 (2.0%)	0 (0.0%)	1.00

ACE inhibitors, Angiotensin-Converting Enzyme inhibitors; ARBs, Angiotensin II Receptor Blockers; CCBs, Calcium channel blockers.

### 3.1 Hemodynamic outcomes and hypertensive crises

**Figure 1:**
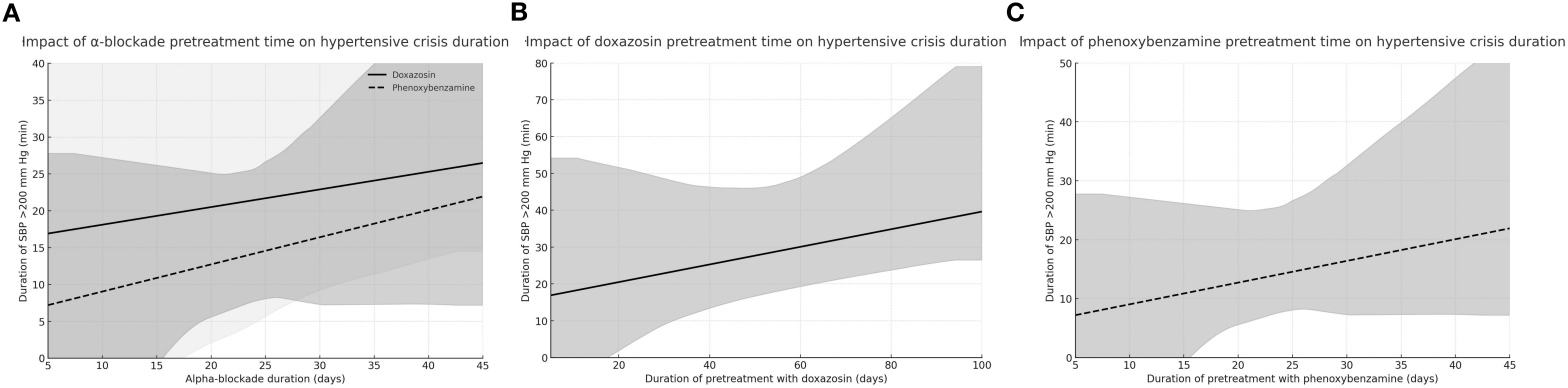
Correlation between hypertensive crisis length (SBP >200 mm Hg) and preoperative alpha-blockade time: **(A)** doxazosin and phenoxybenzamine plotted separately; **(B)** doxazosin only; **(C)** phenoxybenzamine only.

Preoperative systolic blood pressure (SBP) was significantly higher in the DOX group [135.0 (120.0–140.0) mm Hg] compared to the PXB group [125.0 (110.0–140.0) mm Hg; p = 0.03] (**Table 2**) . The frequency of hypertensive crisis, defined as SBP >200 mm Hg, was 23.6% in the PXB group and 32.1% in the DOX group (p = 0.39). However, the duration of hypertensive crisis was significantly longer in the DOX group [15.0 (10.0–30.0) min] compared to the PXB group [10.0 (5.0–15.0) min, p = 0.03]. Prolonged preoperative alpha-blockade was also associated with longer hypertensive crisis episodes in the overall cohort (ρ = 0.77, p < 0.01; **Table 3**; **Figures 1A, B**), although this correlation did not reach statistical significance in the PXB subgroup (**Figure 1C**). No associations were observed between the duration of hypertensive crisis and age, BMI, American Society of Anaesthesiologists (ASA) classification, tumour size, alpha-blocker dose, or metoxycatecholamine concentrations.

**Table 2:** Intraoperative blood pressure fluctuations, vasoactive drug use, and perioperative outcomes.

Variable	DOX (n=55)	PXB (n=55)	p
SBP before surgery (mm Hg), median (IQR)	135.0 (120.0-140.0)	125.0 (110.0-140.0)	0.03
DBP before surgery (mm Hg), median (IQR)	80.0 (70.0-80.0)	80.0 (68.0-80.0)	0.60
SBP 1^st^ measurement during surgery (mm Hg), median (IQR)	110.0 (90.0-120.0)	115.0 (100.0-135.0)	0.40
DBP 1^st^ measurement during surgery (mm Hg), median (IQR)	70.0 (60.0-72.5)	70.0 (55.0-80.0)	0.69
Patients with episodes SBP >200 mmHg, n (%)	17 (32.1%)	13 (23.6%)	0.39
Number of SBP episodes > 200 mm Hg, median (IQR)	0.0 (0.0-1.0)	0.0 (0.0-0.0)	0.28
Duration (min), median (IQR)	15.0 (10.0-30.0)	10.0 (5.0-15.0)	0.03
Patients with episodes SBP >180 mmHg, n (%)	26 (49.1%)	22 (40.0%)	0.44
Number of SBP episodes > 180 mm Hg, median (IQR)	0.0 (0.0-2.0)	0.0 (0.0-1.0)	0.30
Duration (min), median (IQR)	15.0 (10.0-25.0)	10.0 (5.0-20.0)	0.26
Patients with episodes SBP >160 mmHg, n (%)	37 (69.8%)	34 (61.8%)	0.42
Number of SBP episodes > 160 mm Hg, median (IQR)	1.0 (0.0-3.0)	1.0 (0.0-2.0)	0.11
Duration (min), median (IQR)	30.0 (10.0-40.0)	15.0 (6.2-35.0)	0.34
Intraoperative use of vasodilator drugs, n (%)	27 (51.9%)	23 (41.8%)	0.34
Intraoperative use of vasopressive drugs, n (%)	21 (39.6%)	6 (10.9%)	<0.001
Intraoperative use of beta-blockers, n (%)	10 (29.4%)	9 (16.4%)	0.19
Postoperative use of vasopressive drugs, n (%)	17 (30.9%)	8 (14.5%)	0.07
Operative time (min), median (IQR)	90.0 (70.0-125.0)	90.0 (80.0-140.0)	0.18
Anaesthesia duration (min), median (IQR)	130.0 (110.0-162.5)	130.0 (110.0-170.0)	0.65
Complications, n (%)	12 (21.8%)	16 (29.6%)	0.39
Length of hospital stay (days), median (IQR)	3.0 (2.2-4.0)	2.0 (2.0-3.0)	<0.001

SBP, systolic blood pressure; DBP, diastolic blood pressure.

**Table 3:** Correlations between duration of pretreatment with DOX or PXB and perioperative hemodynamics and clinical parameters.

Variable	All group		DOX (n=55)		PXB (n=55)	
	Correlation coefficient	P value	Correlation coefficient	P value	Correlation coefficient	P value
SBP before surgery (mm Hg)	0.12	0.36	0.45	0.16	-0.29	0.18
DBP before surgery (mm Hg)	0.12	0.34	0.09	0.80	0.24	0.27
SBP 1^st^ measurement during surgery (mm Hg)	0.21	0.09	0.48	0.13	0.31	0.15
DBP 1^st^ measurement during surgery (mm Hg)	0.21	0.09	0.40	0.23	0.19	0.39
Number of SBP episodes > 200 mm Hg	0.18	0.15	0.68	0.02	0.04	0.86
Duration (min)	0.77	< 0.01	0.61	0.07	0.63	0.37
Number of SBP episodes > 180 mm Hg	0.24	0.06	-0.58	0.31	0.39	0.06
Duration (min)	0.32	0.08	0.32	0.16	0.20	0.55
Number of SBP episodes > 160 mm Hg	0.28	0.03	-0.03	0.92	0.29	0.18
Duration (min)	0.16	0.31	0.07	0.72	0.34	0.19
Estimated blood loss (ml)	0.0	0.99	0.30	0.43	0.07	0.75
Operative time (min)	0.15	0.27	-0.22	0.51	0.10	0.66
Anaesthesia time (min)	0.16	0.23	0.34	0.37	0.12	0.59
Length of hospital stay (days)	0.06	0.66	0.43	0.19	-0.01	0.95

SBP, systolic blood pressure; DBP, diastolic blood pressure.

Intraoperative vasopressor use was significantly more frequent in the DOX group (39.6% vs. 10.9%, p < 0.001) (**Table 2**). No statistically significant differences were observed in intraoperative vasodilator use or intraoperative use of beta-blockers.

### 3.2 Predictors of intraoperative hypertensive crisis

Univariable analysis showed that diabetes mellitus (OR = 3.59; 1.49–8.63; p = 0.004), urinary metanephrine concentration >10× ULN (OR = 4.36; 1.37–13.94; p = 0.013), and preoperative alpha-blockade >30 days (OR = 3.02; 1.23–7.43; p = 0.016), were significant predictors of hypertensive crisis. In multivariable logistic regression analysis, all three variables remained independent predictors of intraoperative SBP>200 mm Hg: preoperative alpha-blockade >30 days (OR = 3.22; 1.22–8.51; p = 0.018), diabetes mellitus (OR = 2.99; 1.18–7.61; p = 0.021), and urinary metanephrine >10× ULN (OR = 3.89; 1.09–13.95; p = 0.037) (**Table 4**).

**Table 4:** Univariable and multivariable logistic regression analysis of intraoperative hypertensive crisis (SBP >200 mm Hg).

Variable	Univariable OR (95% CI)	Univariable p-value	Multivariable OR (95% CI)	Multivariable p-value
Age at diagnosis (years)	1.02 (0.99–1.05)	0.14	–	–
Sex (female vs. male)	0.68 (0.29-1.58)	0.36	–	–
BMI (kg/m²)	1.03 (0.95–1.12)	0.48	–	–
Diabetes (yes vs no)	3.59 (1.49–8.63)	0.004	2.99 (1.18–7.6)	0.020
ASA class (I–IV)	1.66 (0.80–3.45)	0.18	–	–
Type of alpha-blocker (DOX vs. PXB)	1.53 (0.65–3.56)	0.33	–	–
Dose of phenoxybenzamine (mg)	0.99 (0.97–1.01)	0.39	–	–
Dose of doxazosin (mg)	1.04 (0.97–1.11)	0.26	–	–
Alpha-blockade >30 days	3.02 (1.23–7.43)	0.016	3.22 (1.22–8.51)	0.018
Tumour size (cm)	1.06 (0.78–1.43)	0.72	–	–
Urine metanephrine >10× ULN	4.36 (1.37–13.94)	0.01	3.89 (1.09–13.95)	0.037
Urine normetanephrine >10× ULN	1.38 (0.46–4.07)	0.57	–	–
Urine methoxytyramine >10× ULN	1.79 (0.28–11.26)	0.54	–	–

### 3.3 Duration of the pretreatment and perioperative parameters

In the entire cohort, the duration of preoperative alpha-blockade correlated positively with the number of SBP >160 mm Hg episodes (p = 0.03), (**Table 3**). In the DOX group, longer pretreatment was associated with a greater number of SBP >200 mm Hg episodes (ρ = 0.68, p = 0.02). Longer alpha-blockade was also associated with longer duration of hypertensive crisis (ρ = 0.77, p < 0.01) (**Table 3**, **Figure 1**).

Among patients receiving doxazosin, those who were prepared for more than 30 days (Group C) exhibited a significantly longer duration of SBP >200 mmHg episodes compared to those prepared for 14 days or less (Group A) [30.0 (20.0–42.5) min vs. 10.0 (10.0–15.0) min], p = 0.04), **Supplementary Table S1**). Patients in Group C were more likely to require postoperative vasopressors than those in Group A (30.8% vs. 0.0%, p = 0.05), and had a significantly longer hospital stay [3 (2–4) vs. 2 (2–3) days], p = 0.05. In the PXB group, the number and duration of hypertensive crisis did not differ significantly regardless of the duration of preoperative α-blockade (**Supplementary Table S1**). However, patients in Group C more frequently required postoperative vasopressors compared to Group A (30.0% vs. 0.0%, p = 0.05).

### 3.4 Alpha-blocker dosage and perioperative parameters

Higher doxazosin doses were associated with higher diastolic blood pressure before surgery (ρ = 0.89, p = 0.02), (**Supplementary Table S2**). In the PXB group, a similar association was found (ρ = 0.58, p = 0.003). No statistically significant associations were found between the final alpha-blocker dose and SBP surges, intraoperative hemodynamic parameters, estimated blood loss, operative time, anaesthesia time, or hospital stay.

### 3.5 Postoperative course and clinical outcomes

The proportion of patients requiring vasopressors postoperatively was more than twice in the DOX group in comparison to the PXB group (30.9% vs. 14.5%, p = 0.07) (**Table 2**). Among patients treated with doxazosin, the incidence of postoperative vasopressor use increased with longer duration of preoperative alpha-blockade (**Supplementary Table S1**). While no patients in Group A (1–14 days) required vasopressors, 12.5% of patients in Group B (15–30 days) and 50.0% in Group C (>30 days) received vasopressor support. The overall difference was statistically significant (p = 0.025), with a substantial increase observed in Group C compared to Group A (p = 0.06).

**Figure 2:**
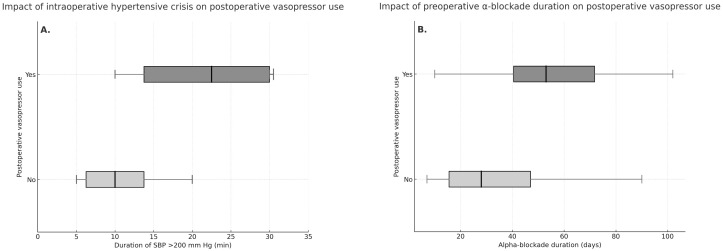
Impact of intraoperative hypertensive crisis **(A)** and preoperative alpha-blockade duration **(B)** on postoperative vasopressor use.

The duration of intraoperative hypertensive crisis (SBP >200 mmHg) was significantly longer in patients who required postoperative vasopressor support [22.5 (13.75-30.00) min] compared to those who did not [10.0 (6.25–13.75) min; p = 0.038] (**Figure 2A**). The duration of preoperative alpha-blockade was also longer in the group who required postoperative vasopressors compared to those who did not require [53 (41–72) vs 28 (16–47) days], p = 0.007 (**Figure 2B**).

The median length of hospital stay was significantly longer in the DOX group [3 (2–4) days] compared to the PXB group [2 (2–3) days, p < 0.001]. The overall complication rate was 25.5%, and among all complications, 71.4% were classified as grade 1–2 according to the Clavien-Dindo classification. The incidence and severity of postoperative complications, including classification based on Clavien-Dindo grading, did not differ significantly between the DOX and PXB groups (all p > 0.20) (**Table 2**). There were eight severe postoperative complications classified as Clavien-Dindo grade 3–5: five occurred in the DOX group and three in the PXB group (p = 0.72). In the DOX group, complications included postoperative haemorrhage requiring surgical intervention, intra-abdominal abscess, respiratory failure necessitating reintubation, severe postoperative ileus, and hemodynamic instability that required prolonged intensive care support. In the PXB group, severe complications consisted of respiratory failure with acute respiratory distress syndrome, intra-abdominal haemorrhage requiring transfusion, and persistent hypotension necessitating vasopressor therapy. There was one death reported in the PXB group. The patient was a 70-year-old woman with significant comorbidities (ASA class IV), diagnosed with an ACTH-producing pheochromocytoma and overt Cushing’s syndrome. On the ninth postoperative day, she developed acute respiratory distress syndrome, which ultimately led to death.

The follow-up duration in the entire cohort was [7.0 (3.5–11.0) years], with shorter follow-up in the DOX group [5.0 (3.0–7.0) years] compared to the PXB group [11.5 (9.0–14.0) years, p < 0.001]. Four cases of tumour recurrence or metastasis were observed (two in each group; p = 1.00). A total of 18 patients died during follow-up (7 in the doxazosin group, 11 in the phenoxybenzamine group), with 17 deaths unrelated to pheochromocytoma. The difference in all-cause mortality between groups was not statistically significant (p = 0.30).

## 4 Discussion

This study is the first to demonstrate that prolonged preoperative alpha-blockade not only does not prevent intraoperative hypertensive crises during pheochromocytoma surgery, but rather substantially raises the risk. Indeed, alpha-blockade lasting more than 30 days emerged as an independent risk factor for intraoperative systolic blood pressure exceeding 200 mmHg. Moreover, extended preoperative alpha-blockade was associated with longer duration of intraoperative hypertensive crisis and more frequent postoperative use of vasopressors. Contrary to the traditional approach recommending dose escalation, no correlation was found between the final titrated dose of either doxazosin or phenoxybenzamine and the incidence or duration of intraoperative hypertensive crises. Doxazosin, when compared with phenoxybenzamine, was found to be related to longer intraoperative hypertensive crises, more frequent perioperative vasopressor use, and prolonged hospital stay.

In the pheochromocytoma surgery, a hypertensive crisis is generally defined as a systolic blood pressure (SBP) that surpasses 200 mm Hg, since SBP levels above this threshold have been proven to be associated with a higher risk of severe intraoperative hemodynamic complications (7–10). The occurrence of a hypertensive crisis, reported in 20% to as many as 60% of cases depending on the institution, raises the risk of intraoperative bleeding, hemodynamic instability, and episodes of perioperative hypotension (10, 18). Over the years, it has been considered that preoperative alpha-blockade should be maintained for no less than 14 days to mitigate the risk of hemodynamic instability, including hypertensive crises (19). However, recent studies have questioned the effectiveness of prolonged preoperative alpha-blockade in preventing perioperative hypertensive crises surgery (13, 14). Kong et al. demonstrated that prolonging the use of doxazosin beyond a duration of 30 days did not provide any further hemodynamic benefits, Conversely, it considerably raised the risk of postoperative hypotension requiring vasopressor treatment and lowered the minimum heart rate during surgery, pointing to a higher rate of bradycardia during surgery (14). Similarly, Yao et al. found that extending phenoxybenzamine administration beyond 14 days did not improve intraoperative hemodynamic stability in patients undergoing pheochromocytoma or paraganglioma surgery (13). Our study reveals that alpha-blockade extended beyond 30 days not only fails to reduce the risk of hypertensive crisis but substantially increases its incidence. Furthermore, we observed a strong positive correlation between the duration of alpha-blockade and the length of hypertensive crisis episodes (ρ = 0.77, p < 0.01), suggesting a cumulative hemodynamic impact of long-term alpha-receptor blockade. Notably, patients who required postoperative vasopressor support not only experienced substantially longer intraoperative hypertensive crises but had also undergone a longer preoperative alpha-blockade, indicating that prolonged alpha-receptor inhibition may impair compensatory mechanisms and contribute to extended hypertensive episodes and increased need for pharmacologic support in the postoperative period. While direct evidence remains limited, a plausible hypothetical mechanism is that prolonged alpha-blockade may lead to adrenergic receptor desensitization, thereby reducing vascular responsiveness to intraoperative catecholamine surges. In addition, sustained vasodilation may contribute to hypovolemia, increasing susceptibility to hypotension following tumour removal and the abrupt drop in circulating catecholamines. Altogether, our study highlights that extended duration of alpha-blockade, rather than providing additional protection, may in fact increase risk of intraoperative hypertensive crisis. These findings underscore the necessity of re-evaluating current perioperative protocols in the management of pheochromocytoma.

Pretreatment with alpha-blockers with gradual dose titration remains the gold standard in the preoperative treatment of pheochromocytoma, aiming to secure adequate adrenergic suppression and to diminish the risk of hemodynamic instability during surgery (2). However, this practice has recently been questioned by Holscher et al., who indicated that neglecting the traditional dose escalation of alpha-blockers did not lead to increased risk of hemodynamic instability and hypertensive crisis. In alignment with these findings, our study revealed that there was no change in the incidence or duration of hypertensive crises with higher final doses of either doxazosin or phenoxybenzamine. Interestingly, in the phenoxybenzamine group we observed a significant positive correlation between final dose and preoperative diastolic blood pressure (Spearman’s ρ = 0.580, p = 0.003), whereas this trend was less evident in patients treated with doxazosin. The mechanisms underlying this association remain unclear; however, phenoxybenzamine, as an irreversible and nonselective alpha antagonist, blocks both α_1_ and presynaptic α_2_ receptors (20). In particular, inhibition of α_2_ receptors has been shown to increase norepinephrine release, which may counteract the vasodilatory effect of α_1_ inhibition by promoting persistent vascular tone through stimulation of β_1_ and β_2_ receptors. Concurrently, sustained vasodilation may activate the renin–angiotensin–aldosterone system, promoting volume retention and elevating diastolic pressure (21). In contrast, doxazosin selectively blocks α_1_ receptors without affecting α_2_, which may explain the weaker link between its dosage and preoperative diastolic pressure in our findings. The observed absence of benefit from higher alpha-blocker doses, along with their potential association with elevated diastolic pressure, as demonstrated in our study and supported by existing literature, suggests that the conventional dose-escalation strategy may warrant reconsideration. In light of our findings, we advocate titrating alpha-blocker doses primarily in patients presenting with well-established risk factors for hypertensive crisis, such as high tumour biochemical activity and comorbidities including diabetes, with the goal of achieving hemodynamic stability and enhancing the safety and effectiveness of perioperative management in pheochromocytoma surgery.

Although the recommended duration of preoperative alpha-blockade is typically 14 days, it is frequently prolonged in clinical practice, as surgery is often scheduled only once normotension has been achieved, which is not always attainable within the limited preoperative period (2). Current guidelines suggest that a target blood pressure of less than 130/80 mmHg in the seated position and greater than 90 mmHg systolic in the upright position, with a heart rate of 60–70 bpm seated and 70–80 bpm standing, is advocated as a standard goal prior to adrenalectomy for pheochromocytoma (20). In line with these recommendations, most physicians strive to achieve these hemodynamic targets before proceeding to adrenalectomy, a process that often necessitates gradual titration of alpha-blockers and, in many instances, the addition of other antihypertensive agents, which ultimately contribute to a prolonged preoperative period (22). Despite this widely accepted approach, whether attaining preoperative blood pressure target levels truly translates into improved surgical outcomes remains uncertain (23–25). Previous research has suggested that elevated preoperative systolic blood pressure may contribute to increased perioperative risk, with studies demonstrating an association between uncontrolled blood pressure and a higher incidence of intraoperative hemodynamic instability, increased blood loss, and postoperative complications (23, 24). In contrast, data from a large retrospective multicentre study involving 2016 patients with pheochromocytoma indicated that the apparent association between hypertension and adverse perioperative outcomes may be attributable to differences in baseline patient characteristics (25). Although hypertensive patients with pheochromocytoma initially appeared to have longer operative time, more frequent use of vasopressors, and a higher overall complication rate compared to normotensive individuals, they were also more likely to be older, have type 2 diabetes, and present with an ASA physical status of III or IV. After controlling for demographic factors both through multivariable logistic regression and propensity score matching, hypertension was no longer independently associated with perioperative complications. These findings suggest that the adverse outcomes initially attributed to hypertension may in fact reflect the influence of other coexisting risk factors, rather than a direct effect of elevated blood pressure itself for operative complications (25).

Selective alpha-1 antagonists such as doxazosin are currently more widely used than phenoxybenzamine in the preoperative management of pheochromocytoma, primarily due to their availability, cost-effectiveness, and convenient once-daily dosing. Nonetheless, a growing body of literature suggests that phenoxybenzamine, the only non-selective and irreversible alpha-blocker, may offer better hemodynamic control during pheochromocytoma surgery (8, 11, 26). Agrawal et al. reported significantly more intraoperative transient severe hypertension (SBP > 220 mmHg) in patients treated with prazosin compared to those receiving phenoxybenzamine (26). Similarly, Kiernan et al. reported that systolic blood pressure elevations exceeding 200 mmHg occurred more frequently in patients undergoing selective alpha-blockade (8). On the other hand, Randle et al. identified a higher occurrence of intraoperative hypotension, defined as mean arterial pressure (MAP) <60 mmHg, as well as a substantially greater need for vasopressor support among patients receiving selective agents (27). Complementary findings were reported by Zhou et al., who showed that doxazosin use was associated not only with an elevated risk of hypertensive and hypotensive episodes, but also with more frequent perioperative administration of vasopressors. Expanding on prior evidence, our findings demonstrate that the duration of hypertensive crises was significantly longer in patients pretreated with doxazosin, even though the overall frequency of such events did not differ significantly between the doxazosin and phenoxybenzamine groups. Notably, in our study, the duration of alpha-blockade was significantly longer in the DOX group; however, this extended preoperative period did not translate into improved intraoperative hemodynamic parameters. Moreover, patients pretreated with doxazosin more frequently required intraoperative vasopressor support and had longer hospital stays, reflecting a higher clinical burden and contributing to increased healthcare costs. These findings align with earlier concerns and underscore that selective blockade may carry a higher risk of sustained hypotension and greater pharmacologic burden. Given the higher risk of perioperative hypotension and greater pharmacologic intervention associated with selective agents, phenoxybenzamine may represent a more appropriate choice in patients with known risk factors for hemodynamic instability, such as diabetes or tumours with markedly elevated catecholamine secretion.

The most commonly used add-on drugs during preoperative preparation for pheochromocytoma are beta-blockers, administered after adequate alpha-blockade in patients with persistent tachycardia. In our cohort, beta-blockers were prescribed in 62% of patients receiving doxazosin and 74% of those receiving phenoxybenzamine (p = ns). Indeed, reported beta-blocker use varies considerably across centres and published series (26, 28–30). Large cohorts from Europe and India have documented beta-blocker administration in 80–100% of patients undergoing adrenalectomy (26, 28, 29), whereas in a recent Chinese study the rate was markedly lower at 24% (30). This heterogeneity likely reflects differences in institutional perioperative pathways and clinical practice traditions, but may also be influenced by ethnic and biological variation in tumour phenotype and catecholamine secretion patterns.

In recent years, several studies have questioned the necessity of routine preoperative alpha-blockade in pheochromocytoma surgery, suggesting that it may not significantly reduce the risk of intraoperative hemodynamic instability and hypertension surges, particularly in patients with small tumours and without markedly elevated metanephrine levels (31, 32). The recent meta-analysis by Wang et al. further strengthened this debate by demonstrating that none of the evaluated intraoperative hemodynamic parameters differed between patients who received α-blockade and those who did not. This included maximum and minimum systolic blood pressure, the frequency of hypertensive and hypotensive instability episodes, and peak heart rate. However, these data originate from nonrandomized studies with considerable risk of bias, substantial heterogeneity, and variable definitions of hemodynamic instability. Moreover, reliably identifying biochemically mild or truly non-functional tumours remains challenging, and hemodynamic instability has been described even in seemingly silent tumours (33, 34). Hence, the question of whether α-blockade can be safely omitted in selected patients remains open, and rigorously designed randomized controlled trials are required to determine which subgroups may not derive benefit from preoperative α-adrenergic blockade (35).

At present, no single universal anaesthetic protocol reliably eliminates intra-operative haemodynamic instability in pheochromocytoma surgery (1, 36–38). A recently reported approach by Mazeh et al. introduced the concept of deliberate compensated vasoplegia, combining continuous non-adrenergic vasodilation with counterbalancing low-dose vasopressor infusion to blunt catecholamine-mediated hypertensive surges (39). In a single-centre cohort, this strategy resulted in near-complete prevention of moderate and severe hypertensive crises, with severe hypertensive events approaching zero and the proportion of intra-operative measurements within the normotensive range increasing from 48% with standard care to 80% with the DCV protocol. Importantly, this regimen relied on proactive continuous infusion therapy rather than reactive bolus treatment, maintaining controlled vasodilation throughout tumour manipulation with tailored vasopressor support to preserve organ perfusion. These findings suggest that a preventative haemodynamic stabilisation strategy may offer superior control compared with traditional intermittent vasoactive titration. This innovative technique represents a promising direction for peri-operative management, although external validation in larger and prospective cohorts is required before broader adoption.

The study has some limitations which should be considered. Our analysis did not reveal a notable effect of the type or duration of blockade on severe morbidity or mortality, which is probably attributable to the limited sample size and the rarity of such events. While this reflects the low incidence of pheochromocytoma, it may reduce statistical power for detecting associations with rare but clinically important outcomes. Although this study includes nearly two decades of consecutive cases from a high-volume tertiary centre, the very low frequency of major complications and the near absence of mortality make it statistically unrealistic for any single-centre cohort to achieve adequate power to compare such rare outcomes. In our series, only isolated severe adverse events occurred, which is consistent with the excellent safety profile reported in other large adrenalectomy cohorts. In our institution’s earlier analysis of more than 500 adrenalectomies, Pędziwiatr et al. documented only one postoperative death, corresponding to a mortality rate of 0.2 percent. Comparable outcomes have been described elsewhere, including the landmark series by Walz et al. in which no perioperative deaths were reported among 161 patients with pheochromocytoma and paraganglioma (40, 41). Under such conditions, differences in complication rates become practically impossible to detect without very large sample sizes. For instance, the observed difference in major complication rates between the two preoperative blockade strategies in our cohort would require approximately 800 patients in each treatment arm to be demonstrated with conventional statistical power. Analyses of mortality would require several thousand patients per group due to the extremely low baseline risk. Given these constraints, evaluation of rare endpoints such as major morbidity or mortality will inevitably require multicentre collaborations or international registry-based studies capable of assembling sufficiently large cohorts.

A further limitation is that the treatment period was longer in the doxazosin group, which may raise the concern that longer preoperative preparation, rather than the drug itself, contributed to longer hypertensive crises. In our cohort, the correlation between pretreatment duration and hypertensive crisis duration was statistically significant only in the overall group (ρ = 0.77, p < 0.01), with a borderline-significant effect in the doxazosin subgroup and a similar positive but non-significant trend in the phenoxybenzamine subgroup. The lack of significance in the phenoxybenzamine group was likely due to fewer events and shorter crisis duration, rather than the absence of an association, indicating that the effect was present in both groups but more pronounced with doxazosin.

Although the administration of vasoactive drugs were determined by specific intra-operative haemodynamic patterns, with vasodilators used for catecholamine-related hypertensive surges during tumour manipulation and vasopressors administered after adrenal vein ligation for hypotension related to catecholamine withdrawal, some variability in the choice and titration of agents may have reflected individual clinician practice (1). This reflects real-world endocrine anaesthesia practice, but the absence of a fully uniform protocol remains a limitation. Current guidelines do not yet provide a single standardised peri-operative algorithm for vasoactive drug management in pheochromocytoma, and the development of consensus-based protocols will be an important goal for future practice. Ultimately, as a retrospective study, it is subject to inherent sources of bias such as selection and information bias, which may influence the observed associations; furthermore, differences in perioperative protocols across institutions may limit the generalizability of our findings to other clinical settings. Despite these limitations, this study offers several important contributions to the current understanding of perioperative management in pheochromocytoma. It is the first to suggest that prolonged alpha-blockade may lead to negative hemodynamic consequences rather than serving a protective role. Moreover, we found no benefit from higher final doses of alpha-blockers in reducing blood pressure surges, which challenges the rationale for traditional dose-escalation protocols. By focusing on clearly defined thresholds for hypertensive crisis, and by analysing detailed alpha-blockade parameters such as type, duration, and dose in relation to both intraoperative and postoperative events, the study adds meaningful insight into a field where evidence remains limited. The fact that all procedures were performed in a single, experienced tertiary centre reduces variability in surgical and anaesthetic management and strengthens the internal consistency of the results. Moreover, prior registration of the study protocol enhances its transparency and methodological credibility.

In conclusion, prolonged preoperative alpha-blockade exceeding 30 days not only failed to reduce the risk of intraoperative hemodynamic instability but was identified as an independent risk factor for hypertensive crisis during pheochromocytoma surgery. Extended blockade was also associated with longer crisis duration, increased need for postoperative vasopressor support, and prolonged hospitalization, particularly among patients receiving selective alpha-1 antagonists. Higher final doses of alpha-blockers offered no additional benefit in stabilizing intraoperative blood pressure. Compared to phenoxybenzamine, doxazosin was linked to less favourable perioperative outcomes, including prolonged hypertensive episodes, more frequent use of vasopressors, and longer hospital stay. These results call into question established assumptions regarding the duration and titration of preoperative alpha-blockade in pheochromocytoma surgery and highlight the need for a critical reassessment of current protocols to reduce the risk of overtreatment and improve patient safety.

## Data Availability

The data analysed in this study is subject to the following licenses/restrictions: Restrictions apply to the availability of some or all data generated or analysed during this study to preserve patient confidentiality or because they were used under license. The corresponding author will on request detail the restrictions and any conditions under which access to some data may be provided. Requests to access these datasets should be directed to michal.pedziwiatr@uj.edu.pl.
