# Supplementary material for: Prolonged alpha-blockade and doxazosin are associated with hypertensive crisis in pheochromocytoma surgery
Source: Front Endocrinol (Lausanne). 2026 Jan 7;16:1682912. doi: 10.3389/fendo.2025.1682912 (PMC12819246; doi:10.3389/fendo.2025.1682912)
Supplement: Supplementary file 1 [file Table1.docx]

**Supplementary Table S1. Perioperative hemodynamics and clinical parameters by duration of alpha-blockade in DOX- and PXB-treated patients (Group A: 1–14 days; B: 15–30 days; C: >30 days)**

| **Variable** | **DOX** | | | | | **PXB** | | | | |
| --- | --- | --- | --- | --- | --- | --- | --- | --- | --- | --- |
|  | Group A | Group B | Group C | Pairwise p-values (A/B, B/C, A/C) | Overall p | Group A | Group B | Group C | Pairwise p-values (A/B, B/C, A/C) | Overall p |
| SBP before surgery (mm Hg) | 120.0 (117.0–130.5) | 140.0 (120.0–142.5) | 140.0 (128.0–155.0) | 0.24 / 0.73 / 0.07 | 0.19 | 140.0 (125.0–140.0) | 131.0 (114.5–140.0) | 120.0 (110.0–125.0) | 0.57 / 0.20 / 0.31 | 0.35 |
| DBP before surgery (mm Hg) | 75.0 (70.0–80.0) | 80.0 (71.5–82.5) | 80.0 (70.0–87.5) | 0.60 / 0.96 / 0.55 | 0.80 | 70.0 (60.0–80.0) | 80.0 (72.8–85.0) | 80.0 (80.0–80.0) | 0.17 / 0.16 / 0.92 | 0.27 |
| SBP 1st measurement during surgery (mm Hg) | 100.0 (82.5–110.0) | 110.0 (87.5–122.5) | 110.0 (100.0–135.0) | 0.55 / 0.44 / 0.13 | 0.27 | 105.0 (85.0–110.0) | 115.0 (100.0–125.0) | 120.0 (115.0–140.0) | 0.34 / 0.14 / 0.42 | 0.28 |
| DBP 1st measurement during surgery (mm Hg) | 60.0 (52.5–60.0) | 65.0 (47.5–72.5) | 70.0 (60.0–80.0) | 0.55 / 0.36 / 0.07 | 0.13 | 62.5 (52.5–68.8) | 60.0 (53.8–70.0) | 75.0 (70.0–100.0) | 1.00 / 0.27 / 0.22 | 0.38 |
| SBP >200 (%) | 1 (16.7%) | 1 (12.5%) | 10 (38.5%) | 0.92 / 0.18 / 0.33 | 0.29 | 1 (16.7%) | 3 (25.0%) | 1 (20.0%) | 1.00 / 1.00 / 1.00 | 0.92 |
| Number of SBP episodes >200 | 0.0 (0.0–0.0) | 0.0 (0.0–0.0) | 0.0 (0.0–1.75) | 0.92 / 0.14 / 0.25 | 0.20 | 0.0 (0.0–0.0) | 0.0 (0.0–0.2) | 0.0 (0.0–0.0) | 0.85 / 0.89 / 1.00 | 0.96 |
| Duration >200 mm Hg (min) | 10.0 (10.0–10.0) | 10.0 (10.0–10.0) | 30.0 (20.0–42.5) | 1.00 / 0.27 / 0.04 | 0.049 | 10.0 (10.0–10.0) | 10.0 (7.5–20.0) | 20.0 (20.0–20.0) | 1.00 / 1.00 / 1.00 | 0.76 |
| SBP >180 | 3 (50.0%) | 3 (37.5%) | 14 (53.8%) | 0.71 / 0.44 / 0.89 | 0.73 | 1 (16.7%) | 7 (58.3%) | 3 (60.0%) | 0.15 / 0.24 / 1.00 | 0.21 |
| Number of SBP episodes > 180 mm Hg | 0.5 (0.0–1.0) | 0.0 (0.0–1.0) | 1.0 (0.0–2.75) | 0.88 / 0.22 / 0.37 | 0.36 | 0.0 (0.0–0.0) | 1.0 (0.0–1.2) | 1.0 (0.0–3.0) | 0.18 / 0.17 / 0.80 | 0.27 |
| Duration >180 mm Hg (min) | 10.0 (7.5–10.0) | 15.0 (8.75–20.0) | 22.5 (15.0–30.0) | 0.66 / 0.31 / 0.07 | 0.13 | 20.0 (20.0–20.0) | 10.0 (7.5–22.5) | 20.0 (12.5–22.5) | 0.66 / 1.00 / 1.00 | 0.87 |
| SBP >160 | 3 (50.0%) | 5 (62.5%) | 21 (80.8%) | 0.71 / 0.31 / 0.13 | 0.25 | 4 (66.7%) | 8 (66.7%) | 4 (80.0%) | 1.00 / 1.00 / 1.00 | 0.85 |
| Number of SBP episodes > 160 mm Hg | 0.5 (0.0–1.0) | 1.0 (0.0–3.25) | 2.0 (1.0–3.0) | 0.41 / 0.41 / 0.04 | 0.12 | 1.0 (0.2–1.8) | 1.0 (0.0–2.2) | 3.0 (1.0–4.0) | 0.77 / 0.26 / 0.42 | 0.50 |
| Duration >160 mm Hg (min) | 10.0 (7.5–12.5) | 17.5 (15.0–45.0) | 30.0 (10.0–45.0) | 0.29 / 0.79 / 0.17 | 0.34 | 10.0 (5.0–21.2) | 20.0 (10.0–40.0) | 35.0 (27.5–40.0) | 0.39 / 0.46 / 0.86 | 0.57 |
| Intraoperative use of β-blockers, n (%) | 1 (16.7%) | 1 (20.0%) | 5 (35.7%) | 1.00 / 0.57 / 0.44 | 0.63 | 2 (33.3%) | 2 (16.7%) | 1 (20.0%) | 0.57/ 1.00 / 1.00 | 0.72 |
| Intraoperative use of vasodilators, n (%) | 2 (33.3%) | 3 (37.5%) | 15 (60.0%) | 0.94 / 0.28 / 0.26 | 0.35 | 2 (33.3%) | 5 (41.7%) | 16 (43.2%) | 0.78 / 0.94 / 0.67 | 0.90 |
| Intraoperative use of vasopressors, n (%) | 1 (16.7%) | 5 (62.5%) | 9 (34.6%) | 0.12 / 0.18 / 0.42 | 0.20 | 2 (33.3%) | 1 (8.3%) | 3 (8.1%) | 0.22 / 0.72 / 0.57 | 0.18 |
| Postoperative use of vasopressors, n (%) | 0 (0.0%) | 1 (12.5%) | 13 (50.0%) | 1.00 / 0.10 / 0.06 | 0.025 | 2 (33.3%) | 1 (8.3%) | 5 (13.5%) | 0.22 / 0.65 / 0.24 | 0.36 |
| Estimated blood loss (ml) | 50.0 (50.0–65.0) | 50.0 (23.75–87.5) | 70.0 (30.0–100.0) | 0.89 / 0.68 / 0.80 | 0.87 | 60.0 (27.5–77.5) | 75.0 (27.5–237.5) | 100.0 (20.0–150.0) | 0.71 / 0.65 / 1.00 | 0.87 |
| Operative time (min) | 95.0 (80.0–127.5) | 90.0 (67.5–110.0) | 110.0 (85.0–140.0) | 0.64 / 0.34 / 0.87 | 0.61 | 65.0 (61.2–91.2) | 97.5 (70.0–131.2) | 80.0 (60.0–130.0) | 0.12 / 1.00 / 0.40 | 0.28 |
| Anaesthesia time (min) | 112.5 (110.0–148.75) | 130.0 (117.5–161.25) | 150.0 (120.0–190.0) | 0.26 / 0.80 / 0.24 | 0.42 | 92.5 (90.0–121.2) | 135.0 (113.8–155.0) | 120.0 (85.0–155.0) | 0.08 / 0.93 / 0.49 | 0.24 |
| Mean length of hospital stay (days) | 1.5 (1.0–2.0) | 2.0 (1.75–2.25) | 2.0 (2.0–3.0) | 0.41 / 0.31 / 0.05 | 0.12 | 3.5 (2.2–4.8) | 3.0 (2.0–3.5) | 4.0 (3.0–4.0) | 0.37 / 1.00 / 0.26 | 0.43 |

Values are reported as median (IQR) for continuous variables or n (%) for binary variables. Statistical significance was assessed using Mann–Whitney U tests for pairwise comparisons and the Kruskal–Wallis test for overall group differences.

**Supplementary Table S2. Correlation between alpha-blocker dose and perioperative hemodynamic and clinical parameters**

| **Variable** | **All group** | | **DOX (n=55)** | | **PXB (n=55)** | |
| --- | --- | --- | --- | --- | --- | --- |
|  | **Correlation coefficient** | **P value** | **Correlation coefficient** | **P value** | **Correlation coefficient** | **P value** |
| SBP before surgery (mm Hg) | -0.07 | 0.56 | 0.12 | 0.82 | 0.14 | 0.52 |
| DBP before surgery (mm Hg) | 0.21 | 0.09 | 0.27 | 0.09 | 0.58 | 0.003 |
| SBP 1st measurement during surgery (mm Hg) | 0.21 | 0.08 | -0.18 | 0.74 | 0.16 | 0.46 |
| DBP 1st measurement during surgery (mm Hg) | 0.22 | 0.08 | -0.20 | 0.70 | 0.28 | 0.19 |
| Number of SBP episodes > 200 mm Hg | -0.02 | 0.89 | 0.36 | 0.48 | 0.08 | 0.70 |
| Duration (min) | -0.19 | 0.49 | 0.13 | 0.80 | -0.56 | 0.32 |
| Number of SBP episodes > 180 mm Hg | 0.06 | 0.64 | 0.42 | 0.41 | 0.14 | 0.51 |
| Duration (min) | 0.09 | 0.63 | 0.28 | 0.59 | -0.35 | 0.29 |
| Number of SBP episodes > 160 mm Hg | -0.07 | 0.57 | -0.17 | 0.74 | -0.18 | 0.40 |
| Duration (min) | 0.18 | 0.23 | 0.20 | 0.70 | 0.10 | 0.71 |
| Estimated blood loss (ml) | 0.06 | 0.67 | 0.09 | 0.86 | 0.03 | 0.91 |
| Operative time (min) | 0.10 | 0.44 | 0.17 | 0.74 | 0.34 | 0.10 |
| Anaesthesia time (min) | 0.11 | 0.37 | -0.06 | 0.91 | 0.35 | 0.10 |
| Mean length of hospital stay (days) | 0.06 | 0.66 | 0.12 | 0.82 | 0.14 | 0.52 |
